# A Green Antioxidant Activity-Integrated Dual-Standard Method for Rapid Evaluation of the Quality of Traditional Chinese Medicine Xuebijing Injection by On-Line DPPH-CE-DAD

**DOI:** 10.1155/2016/2712476

**Published:** 2016-10-31

**Authors:** Jin Li, Jiao Liu, Wei Liu, Xie-an Yu, Jun Cao, Mingrui An, Xiu-mei Gao, Yan-xu Chang

**Affiliations:** ^1^Tianjin State Key Laboratory of Modern Chinese Medicine, Tianjin University of Traditional Chinese Medicine, Tianjin 300193, China; ^2^College of Material Chemistry and Chemical Engineering, Hangzhou Normal University, Hangzhou 310036, China; ^3^Department of Surgery, University of Michigan, Ann Arbor, MI 48109, USA

## Abstract

Much attention has been focused on treatment of sepsis which leads to high mortality all over the world in every year. Antioxidant activity seems to play a prominent role in the treatment of sepsis exhibited by Xuebijing injection. The aim of the present research was to develop an on-line 1, 1-diphenyl-2-picrylhydrazyl- (DPPH-) capillary electrophoresis-diode array detector (on-line DPPH-CE-DAD) method for rapidly assessing antioxidant properties and efficacious material basis of antioxidant activity as a way of quality control of Xuebijing injection. Several parameters affecting the separation were investigated, including the pH and concentrations of buffer, SDS, *β*-CD, and organic modifier as well as voltage and cassette temperature. Compared to previous traditional method, this improved method shortened the experimental cycle and became more efficient because it was successfully applied to analyze total antioxidant activity and contents of twelve antioxidants of Xuebijing injection under the same condition. The results revealed that the on-line DPPH-CE-DAD method was a reagent-saving, rapid, feasible, and green technique for quality control of Xuebijing injection in terms of pharmacological activity and contents of active ingredients. It also offered new opportunities for the analysis of antioxidant activity of complex matrix.

## 1. Introduction

Sepsis, a systemic inflammatory response syndrome caused by infection, is confirmed to be accompanied with the presence of bacteria or highly suspicious focus of infection [1]. Despite the use of antibiotic combination and good supportive therapy and care, treatment for sepsis is still unsatisfactory. The mortality from severe sepsis remains high between 34 and 43% [2]. In a recent septic immunomodulatory study, the traditional Chinese medicine (TCM) attracted much attention for its therapeutic concepts of integration and balanced regulation [3]. Xuebijing (XBJ) injection, extracted from* Carthami flos*,* Paeoniae Radix Rubra*,* Chuanxiong Rhizoma*,* Salviae miltiorrhizae*, and* Angelicae sinensis Radix*, is a traditional Chinese medicine that has been approved for many years by the State Food and Drug Administration (SFDA) of China to clinically treat sepsis [4]. Recent studies has also shown that XBJ is effective for the treatment of serious complications of sepsis, such as hematopoietic injury [4], disseminated intravascular coagulation [5], hematopoietic injury [6], and lung injury [1]. It has been reported that oxidative stress was caused by the pathological process of viral infection and antioxidants could reduce oxidative stress [7, 8]. It has also been demonstrated that Xuebijing injection could decrease the levels of reactive oxygen species (ROS) by increasing glutathione and superoxide dismutase (SOD) levels [4]. Thus, it can be speculated that Xuebijing injection possesses antioxidant properties. However, the underlying material basis of antioxidant activity of Xuebijing injection remains uninvestigated.

Traditional methods of quality control of TCMs included High-Performance Liquid Chromatography (HPLC), Gas Chromatography-Mass Spectrometry (GC-MS), and HPLC-MS [9–11]. Because these methods are limited in determining the contents of compounds, they cannot meet our needs to comprehensively assess the quality of TCMs expected in combination for their pharmacological effect [12]. Therefore, the dual-standard quality assessment was introduced in this study to establish a simple and feasible method to screen the antioxidant components and evaluate the quality of Xuebijing injection.

1, 1-Diphenyl-2-picrylhydrazyl (DPPH) which is a radical-containing compound is usually used to quantify the antioxidant activity of various samples [13, 14]. Various methods were used for the assessment of antioxidants in complex samples, such as the DPPH radical with a spectrophotometer, dot-blot test on a thin-layer chromatography (TLC) plate, and on-line HPLC-DPPH assay [14–16]. These pharmacological methods can lead to long cycle, false positives and can be harmful to the environment. Recently, high-performance capillary electrophoresis (HPCE), as a green separation technology, offers an alternative method that has many advantages compared to other methods, in that it provides good resolution of the sample with a shorter run time, uses less harmful solvents, and is not influenced by color pigments [17, 18].

**Figure 1:**
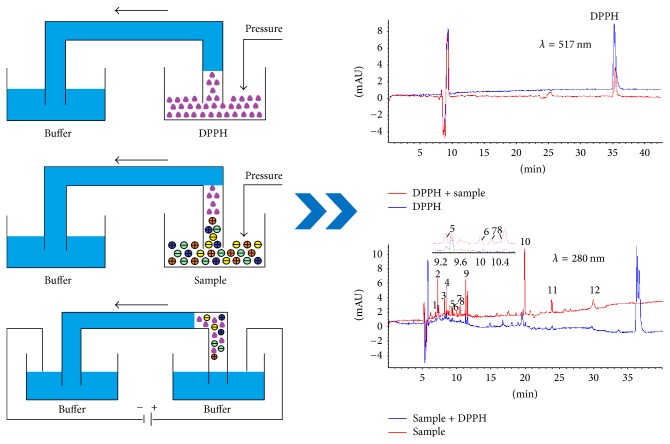
Schematic diagram of on-line DPPH-CE-DAD method.

In a previous paper, we reported our preliminary finding that the on-line DPPH-CE-DAD conditions for determination of the total antioxidant activity and contents of antioxidants were entirely different [18]. If two conditions can be integrated into the same condition, thus the total time needed for the experiment will be significantly cut down and less intensive labor will be achieved. Taking this into account, we have developed a single method that permits the separation and quantification of DPPH and the antioxidants under the same condition by integrating two separation methods into one step. The on-line DPPH-capillary electrophoresis-diode array detector (on-line DPPH-CE-DAD) in a run was selected as a typical example to develop a simple and feasible dual-standard method to screen the antioxidant components and evaluate the quality of Xuebijing (XBJ) injection. The feasibility and precision of on-line DPPH-CE-DAD were discussed in our present report. Schematic diagram of on-line DPPH-CE-DAD method was shown in Figure 1. The improved DPPH-CE-DAD method provided a green, environmental protection and rapid approach to quantitatively analyze total antioxidant activity and contents of antioxidants of Xuebijing injection and other TCMs in short time. An antioxidant activity-integrated dual-standard method by on-line DPPH-CE-DAD will become the advantageous tool for quality control of TCMs.

## 2. Materials and Methods

### 2.1. Chemicals and Reagents

Standard substances including oxypaeoniflorin, hydroxysafflor yellow A, protocatechuic aldehyde, peoniflorin, rosmarinic acid, salvianolic acid B, sodium danshensu, caffeic acid, ferulic acid, senkyunolide I, rutin, and isoquercitrin were purchased from Chengdu Must Bio. Sci. and Tec. Co. Ltd. (Chengdu, China). 10 batches of Xuebijing injections were obtained from Tianjin Chase Sun Pharmaceutical Co. Ltd. (Tianjin, China). Deionized water was provided by a Milli-Q Academic ultra-pure water system (Millipore, Milford, MA, USA). HPLC grade methanol and acetonitrile were obtained from Merck (Germany). DPPH (1, 1-diphenyl-2-picrylhydrazyl) was purchased from Sigma (USA). Other chemicals were of analytical reagent grade. All reagents for capillary electrophoresis were filtrated through 0.22 *μ*m nylon syringe filter.

### 2.2. Preparation of Standard Solutions

Oxypaeoniflorin, hydroxysafflor yellow A, protocatechuic aldehyde, peoniflorin, rosmarinic acid, salvianolic acid B, and sodium danshensu were individually dissolved with deionized water. Caffeic acid, ferulic acid, rutin, and isoquercitrin were individually dissolved with 50% methanol. Senkyunolide I was dissolved with methanol. Appropriate amount of the standards was mixed to prepare a standard solution containing twelve compounds. DPPH solution was dissolved with methanol at a concentration of 1 mg/mL for each day of analysis and stored in the dark prior to use. Standard solutions and samples were stored at 4°C.

### 2.3. Preparation of Quality Control Samples

Quality control (QC) samples of oxypaeoniflorin, hydroxysafflor yellow A, protocatechuic aldehyde, peoniflorin, rosmarinic acid, salvianolic acid B, sodium danshensu, caffeic acid, ferulic acid, senkyunolide I, rutin, and isoquercitrin were prepared by diluting appropriate mixed standard solutions to make three concentration levels (low, medium, and high), respectively.

### 2.4. Preparation of Samples

The Xuebijing injection samples were centrifuged at 14,000 rpm for 10 min. Supernatant was then filtrated through 0.22 *μ*m nylon prior to injection. The different concentration of Xuebijing injection was diluted with deionized water and analyzed by HPCE.

### 2.5. Apparatus and Conditions of DPPH-CE-DAD Method

Capillary electrophoresis was carried out on an Agilent technologies HPCE 7100 (Agilent, Germany) equipped with a diode array detector and a sample tray temperature control system. Agilent ChemStation software for instrumental control and data processing was used. The analysis was performed on an uncoated capillary with effective length of 52 cm and an internal diameter of 50 *μ*m (Ruifeng, Hebei, China). Before the new capillary was initiated, it was flushed with 1.0 M NaOH for 10 min, followed by 0.1 M NaOH for 10 min and deionized water for 10 min. In between each sample throughout the experiment, the capillary was rinsed with 0.1 M NaOH for 3 min, deionized water for 3 min, and buffer for 3 min, successively. The electrolyte buffer was a solution containing 20 mM NaH_2_PO_4_ (pH 5.5), 100 mM sodium dodecyl sulfate (SDS), 10 mM *β*-cyclodextrin (*β*-CD), and 5% ACN (v/v). To estimate the total antioxidant activity and screen antioxidants of Xuebijing injection, hydrodynamic injection was used for sample solution at 50 mbar for 8 s followed by DPPH solution at 50 mbar pressure for 2 s (experimental group). Subsequently, a positive voltage was applied at 25 kV, with a capillary temperature of 22°C. Analytes and DPPH were monitored at 280 nm and 517 nm, respectively. Control group I was defined as Xuebijing injection on-line spiked methanol, and control group II was defined as DPPH solution on-line spiked deionized water.

Consequently, in comparison with control group II, the magnitude of decrease of DPPH peak seen in the electrophoretogram of the experimental group can be used to assess the antioxidant activity of the sample. Similarly, the magnitude of decrease of composition peak seen in the electrophoretogram of the experimental group can be used as a basis of antioxidant activity of Xuebijing injection when compared to control group I.

## 3. Results and Discussion

### 3.1. Optimization of On-Line DPPH-CE-DAD for Determination of Total Antioxidant Activity and Multicomponents of Xuebijing Injection

The electrophoresis system was optimized by adjusting the pH and concentrations of buffer, SDS, *β*-CD, and organic modifier as well as voltage and cassette temperature. The running electrolyte for sample analysis was similar to that described previously [18], except that it was made possible to simultaneously separate and quantify DPPH and the antioxidants in one step by the newly DPPH-CE-DAD method.

**Figure 2:**
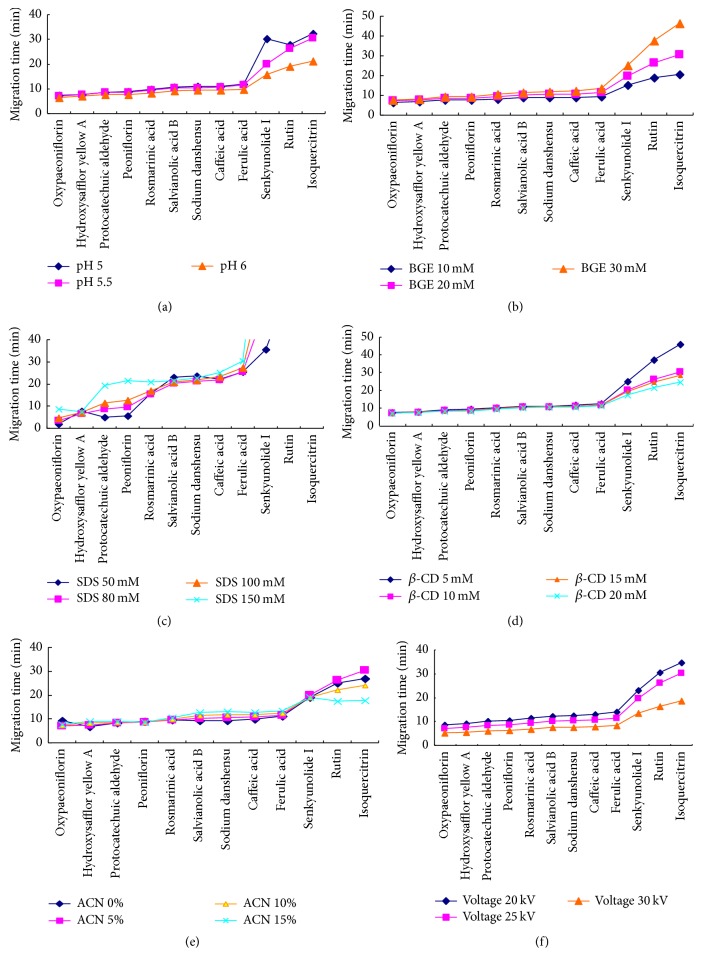
Effects of parameters on the migration time and resolution of twelve peaks: (a) pH of the phosphate buffer, (b) BGE concentration, (c) SDS concentration, (d) *β*-CD concentration, (e) acetonitrile concentration, and (f) separation voltage.

As is known, DPPH is not stable and would produce other substances in acid or alkaline conditions. The pH is the most significant variable influencing the performance of DPPH in comparison with other parameters. Hence, the pH were varied from 5.0 to 8.0 for sample separation while employing a running electrolyte comprising 20 mM NaH_2_PO_4_, 100 mM SDS, 10 mM *β*-CD, and 5% ACN (v/v). When the pH was at 7, 8, and 9, the peak shapes of all compounds were poor. At pH 5.0–6.0, acceptable separation was obtained for all the constituents and prolonged migration time was obtained with decreasing pH of the buffer, revealing that the migration velocity of weak electrolyte and the velocity of the electroosmotic flow (EOF) changed by regulating the pH [19]. From the results, pH of 5.5 was considered satisfactory with respect to migration time (Figure 2(a)).

Ionic strength or concentrations of buffer have significant effects on solute mobility and separation efficiency [20]. NaH_2_PO_4_ buffer at concentrations within the range of 0–40 mM under constant instrumentation conditions was investigated. Comparing the concentration of NaH_2_PO_4_ buffer from 0 to 10 mM which could not reach the baseline separation, 20–40 mM gave a good separation efficiency and resolution for each analyte. However, prolonged migration time was obtained at 30–40 mM as shown in Figure 2(b). The NaH_2_PO_4_ buffer concentration for sample and DPPH determination was set at 20 mM.

The effects of SDS concentrations of 0, 10, 30, 50, 80, 100, and 150 mM for sample separation were tested. As can be seen from Figure 2(c), prolonged migration time was obtained with increase in the concentration of SDS. At low SDS concentrations (0–80 mM), the separation of protocatechuic aldehyde, peoniflorin, salvianolic acid B, sodium danshensu, and caffeic acid was not enough. Increasing the SDS concentration from 80 to 100 mM led to a dramatic improvement in the resolution. When SDS concentration was raised substantially to 150 mM, the separation efficiency of rosmarinic acid and salvianolic acid B was poor as peoniflorin and sodium danshensu. Under 100 mM SDS concentration, there was a good resolution among these compounds.

Addition of *β*-CD with different concentrations (0, 5, 10, 15, and 20 mM) to the running electrolyte was studied. Slightly shortened migration time was observed with increase in the concentration of *β*-CD (Figure 2(d)). When the concentration of *β*-CD was 5 mM, the migration time of salvianolic acid B was the same as sodium danshensu. With the concentration of 10 mM *β*-CD, a baseline resolution of each analyte was observed. Sodium danshensu partially overlapped with caffeic acid at 15 and 20 mM. It may be speculated that *β*-CD with a relative hydrophobic cavity forms inclusion complexes with compounds and the outer hydrophilic layer has been confirmed for the separation of analytes [20].

Organic solvent in the buffer could dissolve insoluble sample and alter hydrophobic analyte-micelle interactions by displacing the analytes from the micelle [21]. Acetonitrile was the most widely used organic modifier in CE [22]. Acetonitrile ranging from 0% to 15% was investigated.  Figure 2(e) showed that the migration time of all analytes shortened when the percentage of acetonitrile added in the buffer was raised. The resolutions of salvianolic acid B and sodium danshensu generally tend to be good from 0% to 5%. An extremely poor resolution resulted when the content of ACN was increased in the running electrolyte exceeding 5%. Therefore, the optimum organic modifier was selected 5% acetonitrile.

With these values established, voltages (25–30 kV) and cassette temperatures (20–25°C) which also influence the peak separation were investigated. A shorter migration time and poor resolution were obtained at a higher voltage (30 kV), while a general increase of migration time was found at a lower voltage (20 kV) (Figure 2(f)). Thus, a positive voltage of 25 kV was chosen as the optimum separation voltage. The cassette temperature was finally set at 22°C.

Based on our previous research [18], 20 mM Na_2_HPO_4_ (pH 6.0) + 50 mM SDS was the optimized condition for analyzing DPPH. However, an optimal condition comprising an electrolyte containing 20 mM NaH_2_PO_4_ (pH 5.5), 100 mM SDS, 10 mM *β*-CD, and 5% ACN (v/v) with the voltage and temperature setting at 25 kV and 22°C was employed in all of our subsequent experiments, thereby reaching a balance between evaluating total antioxidant activity and screening antioxidants of Xuebijing injection in one step.

### 3.2. On-Line Determination of Total Antioxidant Activity of Sample

**Figure 3:**
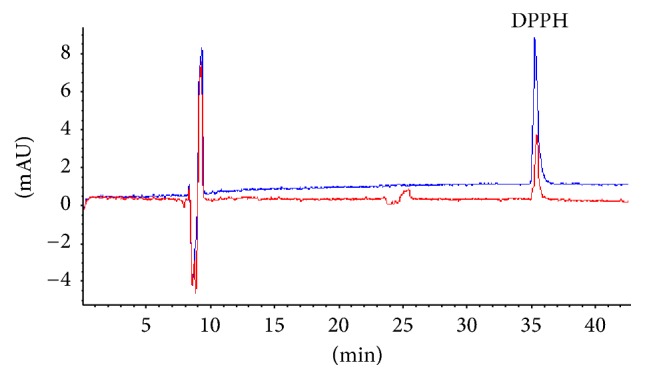
Capillary electropherograms of DPPH: DPPH (blue) and on-line mixed with Xuebijing injection (red). Experimental conditions: 50 *μ*m i.d. × 375 *μ*m o.d. × 60.5 cm length (52 cm effective length), uncoated; 20 mM NaH_2_PO_4_ (pH 5.5), 100 mM SDS, 10 mM *β*-CD, 5% (v/v) ACN, voltage, 25 kV; temperature, 22°C; detection wavelength, 517 nm; pressure injection, 50 mbar for 2 s.

DPPH assay based on the reduction of absorbance at 517 nm of the stable DPPH radical by an antiradical is easy and potentially accurate for measuring the general radical scavenging capabilities of antioxidants [23]. Relying on the proposed DPPH-CE-DAD method as described above, the absorbance of DPPH on-line spiked Xuebijing injection (experimental group) was relatively weak compared to that of the on-line spiked deionized water (control group I) because DPPH would react with antioxidants of Xuebijing injection in the capillary after injecting separately (Figure 3). The relative percentage of inhibition of DPPH was determined by the following equation: [Inhibition  (%) = (*P*_0_ − *P*_1_)/*P*_0_ × 100%]. *P*_0_ was peak area of DPPH (on-line spiked deionized water) and *P*_1_ was peak area of DPPH (on-line spiked Xuebijing injection). In the total antioxidant activity assay of samples, Xuebijing injections with different diluted times were on-line spiked with DPPH, respectively, to carry out the maximal inhibitory concentration at 50% (IC_50_) which denotes the diluted times of sample required to scavenge 50% of DPPH radicals.  Table 1 displayed the IC_50_ values of 10 batches of Xuebijing injections. Results illustrated that Xuebijing injections have antioxidant activity and there was a significant difference in the IC_50_ values of each batch which could speculate that the discrimination of activity was as a result of different contents of antioxidants.

### 3.3. Method Validation

The calibration graphs of twelve antioxidants of Xuebijing injection were established with the peak area ratio as ordinate (*y*) versus the concentration in *μ*g/mL as abscissa (*x*). They were obtained over the range of 33.3–400 *μ*g/mL, 25–600 *μ*g/mL, 4.2–50 *μ*g/mL, 62.5–2000 *μ*g/mL, 5.8–70 *μ*g/mL, 10–120 *μ*g/mL, 13–156 *μ*g/mL, 2.5–30 *μ*g/mL, 2.5–60 *μ*g/mL, 20–480 *μ*g/mL, 13–156 *μ*g/mL, and 11–132 *μ*g/mL for oxypaeoniflorin, hydroxysafflor yellow A, protocatechuic aldehyde, peoniflorin, rosmarinic acid, salvianolic acid B, sodium danshensu, caffeic acid, ferulic acid, senkyunolide I, rutin, and isoquercitrin, respectively. The straight lines of the twelve compounds obtained from six separate experiments had good correlation coefficients. The regression equations and their correlation coefficients were *y* = 0.0658*x* + 0.3049 (*R* ^2^ = 0.9991), *y* = 0.3099*x* − 1.1776 (*R* ^2^ = 0.9999), *y* = 0.6055*x* − 0.2398 (*R* ^2^ = 0.9996), *y* = 0.1948*x* − 0.6145 (*R* ^2^ = 0.9998), *y* = 0.3097*x* − 0.0843 (*R* ^2^ = 0.9990), *y* = 0.2089*x* − 0.526 (*R* ^2^ = 0.9995), *y* = 0.1248*x* − 0.311 (*R* ^2^ = 0.9982), *y* = 0.8707*x* − 0.2758 (*R* ^2^ = 0.9995), *y* = 0.8666*x* − 0.3651 (*R* ^2^ = 0.9995), *y* = 0.198*x* − 0.8145 (*R* ^2^ = 0.9993), *y* = 0.2938*x* − 1.0819 (*R* ^2^ = 0.9990), *y* = 0.3888*x* − 0.0695 (*R* ^2^ = 0.9991), respectively. The limit of detection (LOD) and the limit of quantitation (LOQ) were described as the sample concentrations gave rise to a signal-to-noise (S/N) ratio of 3 and 10, respectively.  Table 2 showed the LODs and LOQs of oxypaeoniflorin, hydroxysafflor yellow A, protocatechuic aldehyde, peoniflorin, rosmarinic acid, salvianolic acid B, sodium danshensu, caffeic acid, ferulic acid, senkyunolide I, rutin, and isoquercitrin.

The precision and accuracy of the proposed method was evaluated on the basis of peak area expressed as relative standard deviation (RSD). The intraday and interday precisions at three typical assay concentrations of each constituent and DPPH were evaluated for six replicates within one day and over three successive days, respectively. The intra- and interday accuracies of twelve antioxidants were within the range of 92.1%–105%. The RSDs of mean precision of twelve antioxidants were below 5.1% for intraday and 4.9% for interday, respectively (Table 3). The RSDs for both intra- and interday of DPPH were 2.4% and 0.9%, respectively. The recoveries of each component were calculated from the calibration graph constructed from the sample spiked with an equivalent amount of the standard solution. The results of recoveries of twelve compounds listed in Table 2 were all in the range of 97.4%–102% and the RSDs were below 4.8%.

The stability expressed by peak area was evaluated by injecting repetitive quality control samples of twelve compounds at low, medium, and high concentrations and DPPH in the CE equipment over 24 h. Table 3 showed that the accuracies of antioxidants were within the range of 94.3%–104% and the RSDs of all compounds and DPPH were below 8.5%, respectively. The result confirmed that they were stable for 24 h at 4°C.

All these data indicated that electrophoretic assay method was acceptable and could be applied in determining the total antioxidant activity of the multicomponents of Xuebijing injection.

### 3.4. On-Line Screening of Antioxidants of Sample

**Figure 4:**
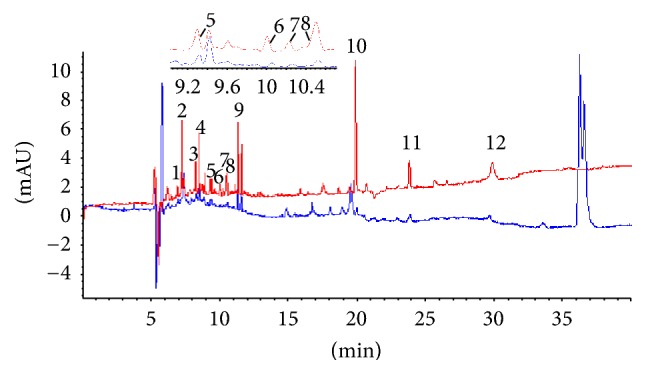
Capillary electropherograms of Xuebijing injection: Xuebijing injection (red) and on-line mixed with DPPH (blue). Peaks: 1 = oxypaeoniflorin, 2 = hydroxysafflor yellow A, 3 = protocatechuic aldehyde, 4 = peoniflorin, 5 = rosmarinic acid, 6 = salvianolic acid B, 7 = sodium danshensu, 8 = caffeic acid, 9 = ferulic acid, 10 = senkyunolide I, 11 = rutin, and 12 = isoquercitrin. Experimental conditions: 50 *μ*m i.d. × 375 *μ*m o.d. × 60.5 cm length (52 cm effective length), uncoated; 20 mM NaH_2_PO_4_ (pH 5.5), 100 mM SDS, 10 mM *β*-CD, 5% (v/v) ACN, voltage, 25 kV; temperature, 22°C; detection wavelength, 280 nm; pressure injection, 50 mbar for 8 s.

Although the antioxidant activity of Xuebijing injection has been demonstrated, it was not clear as to which component was responsible for the antioxidant activity. Taking the identification of active ingredients into account, the peak area of all of the ingredients of Xuebijing injection was compared successively between sample on-line spiked DPPH (experimental group) and sample on-line spiked methanol (control group II) (Figure 4). The components examined from the decreased peak area were compared with reference standard to facilitate the identification and confirmation of the antioxidants of Xuebijing injection, including oxypaeoniflorin, hydroxysafflor yellow A, protocatechuic aldehyde, peoniflorin, rosmarinic acid, salvianolic acid B, sodium danshensu, caffeic acid, ferulic acid, senkyunolide I, rutin, and isoquercitrin (Figure 4). The newly on-line DPPH-CE-DAD method facilitated the rapid screening of major antioxidants of TCMs and was meaningful for further quality assessment using key selected markers.

### 3.5. Sample Analysis

**Figure 5:**
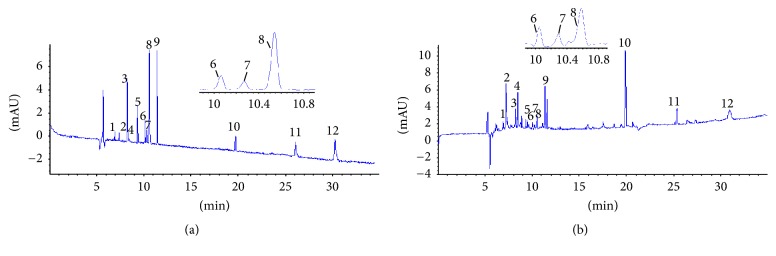
Capillary electropherograms of standard mixture of twelve compounds (a) and Xuebijing injection (b). Peaks: 1 = oxypaeoniflorin, 2 = hydroxysafflor yellow A, 3 = protocatechuic aldehyde, 4 = peoniflorin, 5 = rosmarinic acid, 6 = salvianolic acid B, 7 = sodium danshensu, 8 = caffeic acid, 9 = ferulic acid, 10 = senkyunolide I, 11 = rutin, 12 = isoquercitrin. Experimental conditions: 50 *μ*m i.d. × 375 *μ*m o.d. × 60.5 cm length (52 cm effective length), uncoated; 20 mM NaH_2_PO_4_ (pH 5.5), 100 mM SDS, 10 mM *β*-CD, 5% (v/v) ACN, 25 kV; temperature, 22°C; detection wavelength, 280 nm; pressure injection, 50 mbar for 8 s.

The developed DPPH-CE-DAD method was applied for analyzing the contents of twelve antioxidants of 10 batches of Xuebijing injection under the optimized conditions.  Figure 5 displayed the typical chromatographic profile of the sample. The results of the sample analysis for oxypaeoniflorin, hydroxysafflor yellow A, protocatechuic aldehyde, peoniflorin, rosmarinic acid, salvianolic acid B, sodium danshensu, caffeic acid, ferulic acid, senkyunolide I, rutin, and isoquercitrin were in the range of 39.1–54.6 *μ*g/mL, 381.2–529.4 *μ*g/mL, 15.4–27.3 *μ*g/mL, 1736.6–1980.9 *μ*g/mL, 10.8–25.4 *μ*g/mL, 6.9–17.4 *μ*g/mL, 13.1–24.7 *μ*g/mL, 9.4–14.6 *μ*g/mL, 32.0–37.0 *μ*g/mL, 266.1–382.4 *μ*g/mL, 18.8–28.0 *μ*g/mL, and 26.7–40.2 *μ*g/mL, respectively, which illustrated major difference in the concentrations of twelve antioxidants of each batch of Xuebijing injection (Table 4).

**Figure 6:**
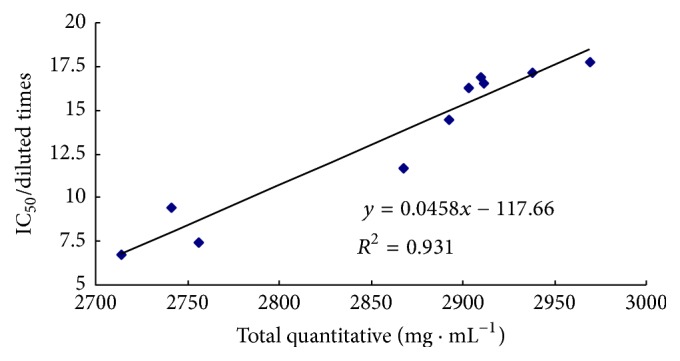
Relationship of the total quantitative and antioxidant activity of Xuebijing injection.

Given the difference in the IC_50_ values of 10 batches, it was likely to be as a result of the different contents of antioxidants. As shown in Figure 6, the data from total content of twelve compounds with antioxidant activity across 10 batches of Xuebijing injections showed a good correlation (*R* ^2^ = 0.931) with above-referred data of determination of total antioxidant activity of samples. The result illustrated that twelve selected antioxidants can serve as key quality control markers to maintain batch-to-batch uniformity and efficacy. It was also validated that the proposed method was feasible and accurate in determining the total antioxidant activity with multiple active ingredients for quality control of TCMs.

## 4. Conclusions

To improve the previous DPPH-CE-DAD method, we developed a simple method that made it possible to separate and quantify DPPH and the antioxidants under the same condition. In the course of on-line mixing DPPH and sample, the magnitude of decrease of DPPH peak seen in the electrophoretogram could calculate the total antioxidant activity of Xuebijing injection. Similarly, twelve antioxidant active ingredients containing oxypaeoniflorin, hydroxysafflor yellow A, protocatechuic aldehyde, peoniflorin, rosmarinic acid, salvianolic acid B, sodium danshensu, caffeic acid, ferulic acid, senkyunolide I, rutin, and isoquercitrin were rapidly screened relying on the magnitude of decrease of composition peak seen. Results illustrated that the advantages of this developed method were reagent-saving, rapid, feasible, and green technique which is not harmful to the environment. The improved on-line DPPH-CE-DAD method achieved the goal in analyzing the total antioxidant activity and contents of antioxidants of Xuebijing injection in one step and was applicable to evaluate the quality of TCMs which exerted directly their antioxidant effects on the radical itself. Furthermore, this presented approach can be applied in evaluating the antioxidant activity of unknown molecules.

## Figures and Tables

**Table 1:** The result of IC_50_ values of 10 batches of samples (*n* = 3).

Sample	IC_50_ (*μ*g/mL)
1301291	16.3
1303041	14.5
1303051	17.2
1303071	16.6
1302221	9.40
1302231	16.8
1303021	17.7
1303042	11.7
1303031	6.70
1302051	7.40

**Table 2:** The calibration curves, linearity ranges, LODs, LOQs, and recoveries of twelve compounds (*n* = 6).

Compounds	Regression equation	*R* ^2^	Linearity range (*μ*g/mL)	LOD (*μ*g/mL)	LOQ (*μ*g/mL)	Recovery	
						Average (%)	RSD (%)
Oxypaeoniflorin	*y* = 0.0658*x* + 0.3049	0.9991	33.3–400	10.0	32.0	101.1	4.6
Hydroxysafflor yellow A	*y* = 0.3099*x* − 1.1776	0.9999	25–600	1.2	3.8	97.4	2.9
Protocatechuic aldehyde	*y* = 0.6055*x* − 0.2398	0.9996	4.2–50	1.2	4.1	100.1	3.5
Peoniflorin	*y* = 0.1948*x* − 0.6145	0.9998	62.5–2000	1.5	5.0	98.2	1.4
Rosmarinic acid	*y* = 0.3097*x* − 0.0843	0.9990	5.8–70	1.5	5.6	101.1	3.7
Salvianolic acid B	*y* = 0.2089*x* − 0.526	0.9995	10–120	3.0	9.4	100.1	4.5
Sodium danshensu	*y* = 0.1248*x* − 0.311	0.9982	13–156	4.0	13.0	99.7	4.8
Caffeic acid	*y* = 0.8707*x* − 0.2758	0.9995	2.5–30	0.8	2.5	99.3	4.8
Ferulic acid	*y* = 0.8666*x* − 0.3651	0.9995	2.5–60	0.8	2.5	101.9	2.7
Senkyunolide I	*y* = 0.198*x* − 0.8145	0.9993	20–480	5.0	17.4	97.7	3.9
Rutin	*y* = 0.2938*x* − 1.0819	0.9990	13–156	4.0	13.0	99.5	3.9
Isoquercitrin	*y* = 0.3888*x* − 0.0695	0.9991	11–132	3.5	11.0	102.3	2.5

**Table 3:** Intraday and interday accuracy and precision and stability of twelve compounds and DPPH (*n* = 6).

Compounds	Concentrations (*μ*g/mL)	Intraday		Interday		Stability for 24 h	
		Accuracy (%)	RSD (%)	Accuracy (%)	RSD (%)	Remains (%)	RSD (%)
Oxypaeoniflorin	66.7	94.9	3.4	97.8	2.8	97.2	3.7
	133.3	97.6	2.3	97.5	0.5	99.9	3.4
	400	96.6	2.7	98.1	1.4	96.5	0.9

Hydroxysafflor yellow A	50	97.8	1.1	97.5	0.2	100.9	4.5
	100	92.1	4.1	97.0	4.9	95.0	4.7
	300	96.7	5.1	98.4	2.3	97.1	0.8

Protocatechuic aldehyde	8.3	103.7	4.9	97.8	4.9	97.4	8.5
	16.7	97.4	2.6	100.3	2.8	99.4	3.0
	50	100.6	0.9	98.5	3.1	101.3	0.9

Peoniflorin	166.7	102.1	0.4	98.7	3.0	101.9	0.3
	333.3	100.6	0.8	98.6	1.9	101.5	1.3
	1000	101.8	0.2	99.2	2.7	103.4	2.2

Rosmarinic acid	11.7	95.8	3.7	98.8	4.5	95.6	0.2
	23.3	102.2	3.5	102.8	1.5	102.6	0.6
	70	101.9	3.0	102.3	0.4	101.9	0.6

Salvianolic acid B	20	96.5	4.0	98.2	1.7	97.7	2.0
	40	98.7	2.9	96.1	2.2	99.2	0.8
	120	98.4	4.0	96.7	4.1	98.0	0.4

Sodium danshensu	26	101.6	4.4	101.7	0.2	103.6	2.9
	52	104.7	2.4	104.2	1.3	103.7	1.3
	156	102.9	3.6	101.5	1.1	102.1	1.0

Caffeic acid	5	105.2	4.5	99.5	4.6	103.9	1.5
	10	100.2	2.8	96.5	3.6	98.9	1.8
	30	97.8	2.8	97.2	1.2	97.7	0.3

Ferulic acid	5	102.0	4.7	100.4	3.7	100.3	2.0
	10	100.7	2.8	97.7	3.1	101.9	1.8
	30	94.4	1.7	97.0	2.5	97.4	4.5

Senkyunolide I	40	99.2	3.9	100.3	2.8	97.9	1.7
	80	101.4	2.3	100.2	1.3	101.7	0.4
	240	100.7	0.8	101.8	0.9	101.2	0.7

Rutin	26	103.0	3.6	101.3	1.4	100.1	3.8
	52	97.0	1.5	97.8	0.8	94.3	4.0
	156	99.7	2.2	97.5	2.5	99.5	0.3

Isoquercitrin	22	100.0	3.3	97.4	2.2	97.8	3.0
	44	100.7	4.9	100.2	1.3	99.8	1.0
	132	104.2	1.7	100.6	3.6	101.4	3.7

DPPH	1000	—	2.4	—	0.9	—	1.4

**Table 4:** Contents of twelve compounds in 10 batches of samples.

Samples	Oxypaeoniflorin	Hydroxysafflor yellow A	Protocatechuic aldehyde	Peoniflorin	Rosmarinic acid	Salvianolic acid B	Sodium danshensu	Caffeic acid	Ferulic acid	Senkyunolide I	Rutin	Isoquercitrin
1301291	49.4	381.2	15.7	1962.9	19.1	17.4	21.6	12.4	36.5	332.4	23.0	32.0
1303041	44.2	482.2	24.5	1806.2	16.9	11.0	22.6	9.5	33.9	382.4	26.6	32.7
1303051	39.3	466.7	15.4	1892.3	15.7	18.4	22.1	9.4	36.7	362.3	28.0	32.1
1303071	39.1	456.2	13.9	1894.1	14.1	12.6	17.9	9.4	34.8	368.3	18.9	32.3
1302221	39.3	529.4	19.2	1736.6	14.0	18.4	24.7	7.4	33.2	266.1	20.9	32.3
1302231	40.9	440.2	21.3	1899.8	16.6	13.9	23.6	9.6	35.3	352.0	23.8	32.6
1303021	48.1	415.4	21.1	1980.9	15.2	11.94	13.7	11.4	32.0	369.3	18.8	31.8
1303042	46.3	426.6	27.3	1881.6	25.4	12.2	23.5	14.0	37.0	312.6	21.2	40.2
1303031	54.6	389.9	24.1	1751.2	10.8	6.9	13.1	9.7	34.6	371.5	21.2	26.7
1302051	46.1	405.7	24.3	1840.6	18.4	13.3	19.7	14.6	34.8	280.9	21.1	36.6
